# Epibulbar complex and osseous choristoma: Clinicopathological study with interesting associations

**DOI:** 10.1016/j.amsu.2018.10.027

**Published:** 2018-10-31

**Authors:** Mohammed M. Aldossary, Hind M. Alkatan, Azza MY. Maktabi

**Affiliations:** a Ophthalmology Department, Prince Sultan Military Medical City, Riyadh, Saudi Arabia; b Anterior Segment Division, King Khaled Eye Specialist Hospital, Riyadh, Saudi Arabia; c Departments of Ophthalmology & Pathology, King Saud University, Riyadh, Saudi Arabia; d Pathology & Laboratory Medicine Department, King Khaled Eye Specialist Hospital, Riyadh, Saudi Arabia

## Abstract

**Materials and methods:**

A retrospective cohort study of cases with tissue diagnosis of epibulbar choristoma in 2 centers presenting during the period: 2000–2016 with focus on cases of complex and osseous choristoma. Demographic and clinical data were collected by the resident from medical records. The histopathological slides were reviewed by 2 pathologists and cases of complex and osseous choristoma were analyzed by biostatical staff.

**Results:**

120 epibulbar choristomas were identified. 13/120 patients (10.8%) with complex choristoma and 2/120 patients (1.7%) with osseous choristoma. 15 cases were further analyzed: 7 were males and 8 were females. Mean age at presentation was 7.6 year. Commonest location was temporal in 66.6%. The presence of smooth muscle component was associated with a larger size choristoma (p = 0.042). 73.3% had other ophthalmic manifestations (mostly eyelid anomalies) while 9 systemic associations (mostly Goldenhar's syndrome) were found in 8/13 cases of complex choristoma.

**Conclusion:**

Epibulbar complex and osseous choristomas are rare. Histopathologically, the presence of smooth muscle significantly correlates with the lesion size. Complex choristoma is more likely to be associated with systemic manifestations. Further genetic studies for this condition are recommended.

## Highlights

• Choristomas are congenital tumors that may affect the epibulbar area.
• They arise from ectopic pluripotent cells differentiating into simple or complex choristoma.
• We present our cases to highlight interesting ophthalmic and/or systemic associations.
• This might open the door for future molecular genetic testing.
• Significant association is found between smooth muscle component and larger size choristoma.

## 1 Introduction

Choristomas or heterotopias are defined as congenital lesions composed of normal cells or tissue occupying an abnormal location. Epibulbar choristoma is considered a rare condition with a prevalence ranges from 1 in 10,000 to 3 in 10,000, although it is the most common form of epibulbar tumors in pediatric patients [1,2]. Ocular choristoma could affect different structures such as the eyelid, iris, retina, choroid and in the case of epibulbar choristoma, it appears in the cornea, limbus, and the conjunctival space, where studies showed that most of the lesions in this area are choristomas [3]. In terms of extent, choristomas varies from small flat lesion to immense tumours which intensively occupy the whole epibulbar area [1,4]. Epibulbar choristoma can be defined as simple when the ectopic pluripotent cells differentiate into single tissue. However, reports showed a complex nature of choristomas when cells even arises from 2 germ layers, the ectoderm and mesoderm differentiate into a variety of tissues as skin, bone, lacrimal gland, cartilage, or infrequently to myxomatous tissue [[5], [6], [7]].

Epibulbar choristomas are normally defined as sporadic and they can develop alone, nevertheless, they can occasionally appear combined with different syndromes, such as Goldenhar's syndrome, epidermal nevus syndrome, encephalo-cranio cutaneous lipomatosis (ECCL), oculo-cerebro-cutaneous syndrome, and oculodermal syndrome [8,9].

The current work represents our experience with choristomatous lesions from the clinical and histopathological aspects to highlight important ophthalmic and/or systemic associations that might open the door for future molecular genetic testing. We also analyzed the histopathological constituents of epibulbar complex choristoma to reveal any significant relevance of each possible component to the clinical features, mainly the average size and the location of the lesion.

### 1.1 Patients and methods

This study was prepared in accordance with the ethical standards of the human ethics committee at KKESH and approval from the Research department in accordance with the Helsinki Declaration with the number (RP-1664-R). A general informed consent was taken from all cases which includes permission for anonymous use of photos and reporting. All histologic slides for cases presenting with an epibulbar mass and diagnosed histopathologically as a choristoma (dermoid, dermolipoma, complex and osseous) at xxx University hospital (KAUH) and xxxx Hospital (KKESH) between 1st of January 2000 and 31st of December 2016 were collected for initial review to be able to confirm the diagnosis and calculate the prevalence of complex/osseous choristoma among all choristomas for further studying. Cases where the choristomatous lesion was identified upon histopathological review as a simple limbal dermoid/dermolipoma were further excluded. The clinical data of the corresponding patients with confirmed tissue diagnosis of complex/osseous epibulbar choristoma in all age groups (from birth) was collected using a specially designed Data collection sheet consisting of 2 major parts:

1 Demographic/clinical: This part included age at presentation, gender, family history of similar conditions, associated syndromes: Goldenhar's, organoid nevus syndrome or others, any ocular association, location of choristoma, lesion size at presentation, progression of size, any other choristoma in the body, type of surgical intervention (incisional biopsy/excisional biopsy), follow up period and clinical evidence of recurrence.
2 Histopathological: This part included confirmation of the diagnosis, the detailed histopathological features, the types of tissue seen and their estimated proportion.

All cases of simple choristoma where excluded from further analysis since they do not match the research question(s) and the purpose of the study. For the statistical analysis, our data was collected and entered in a spreadsheet with the use of Microsoft Excel 2010^®^ software. Coding and management of the data were performed using Excel then analyzed using *StatsDirect*^®^ statistical software, version *2.7.2* (*StatsDirect* Ltd., Cheshire, UK) and SPSS^®^ version 20.0 (*IBM* Inc.^,^ Chicago, Illinois, USA).

Variables were categorized first for descriptive analysis then frequencies, percentages and continuous variables were presented in the form of mean (±Standard Deviation) and range (minimum to maximum). The means for each two categories of data based on the relationship between the different types of tissues and variables (average size of choristoma, location and presence of systemic association) were compared using Mann Whitney test. Any output with A finding of a *p* value below 0.05 was considered as sign of statistical significance.

Furthermore, clinicopathological correlation was presented with review of the relevant English-written literature.

This work has been conducted and prepared for publication in accordance with the STROCSS guideline (Strengthening the Reporting of Cohort Studies in Surgery) [10].

## 2 Results

During a 16-year period, there were 120 patients with epibulbar choristoma, mostly being simple dermoid/dermolipoma except for 15 patients with complex/osseous choristoma accounting for 12.5% of the total number. These were distributed as 13/15 patients with complex choristoma (10.8%), and 2/15 patients with osseous choristoma (1.7%). All cases studied further were from Saudi Arabia. Out of the 15 patients, 7 cases (46.7%) were males and 8 cases (53.3%) were females. Mean age at the time of presentation was 7.6 years. The mean duration of patients’ complaint was 83.4 months, however, the epibulbar mass was noticed since birth in 13 cases (86.7%) and was increasing progressively in size in 40.0%. Decreased vision was reported in 4 cases (26.7%) and there were no other significant complaints such as pain or diplopia.

The most common location of the epibulbar mass was temporal in 10 cases (66.6%). In one case, the whole globe was enucleated later at the time of study for another associated ocular condition and the exact location was not clearly identified in the chart. The remaining 4 cases were located as follows: 2 cases nasally and 2 cases superiorly.

Ophthalmic associated manifestations were documented in 11/15 cases (73.3%) as summarized in Table 1 with the commonest being lid abnormalities followed by optic nerve abnormalities. Upper Lid coloboma by itself was the most common association in one third of the cases.

**Table 1:** Associated ophthalmic manifestations in 11 cases (complex/osseous choristoma).

Structure	Abnormality	Number	Associated syndrome(s)	Total
Eyelid	Coloboma	5	2 cases had Goldenhar's syndrome 1 case had LNSS	6
	Ptosis	1	None	
Optic nerve	Coloboma	1	None	3
	Hypoplasia	1	Goldenhar's syndrome/ECCL	
	Peripapillary hypopigmentation	1	_	
Retina	Coat's disease	1	_	2
	RPE atrophy	1	_	
Choroid	Calcification	1	LNSS	1
Cornea	Microcornea	1	Goldenhar's syndrome/ECCL	1
NLD	NLD obstruction	1	Goldenhar's syndrome	1
EOM	Limitation of up-gaze	1	LNSS	1
Globe	Dystopia	1	LNSS	1

LNSS: linear nevus sebaceous syndrome; ECCL: encephalo-cranio-cutaneous lipomatosis; RPE: Retinal pigmented epithelium; NLD: Nasolacrimal duct, EOM: Extra-ocular motility.

**Fig. 1:**
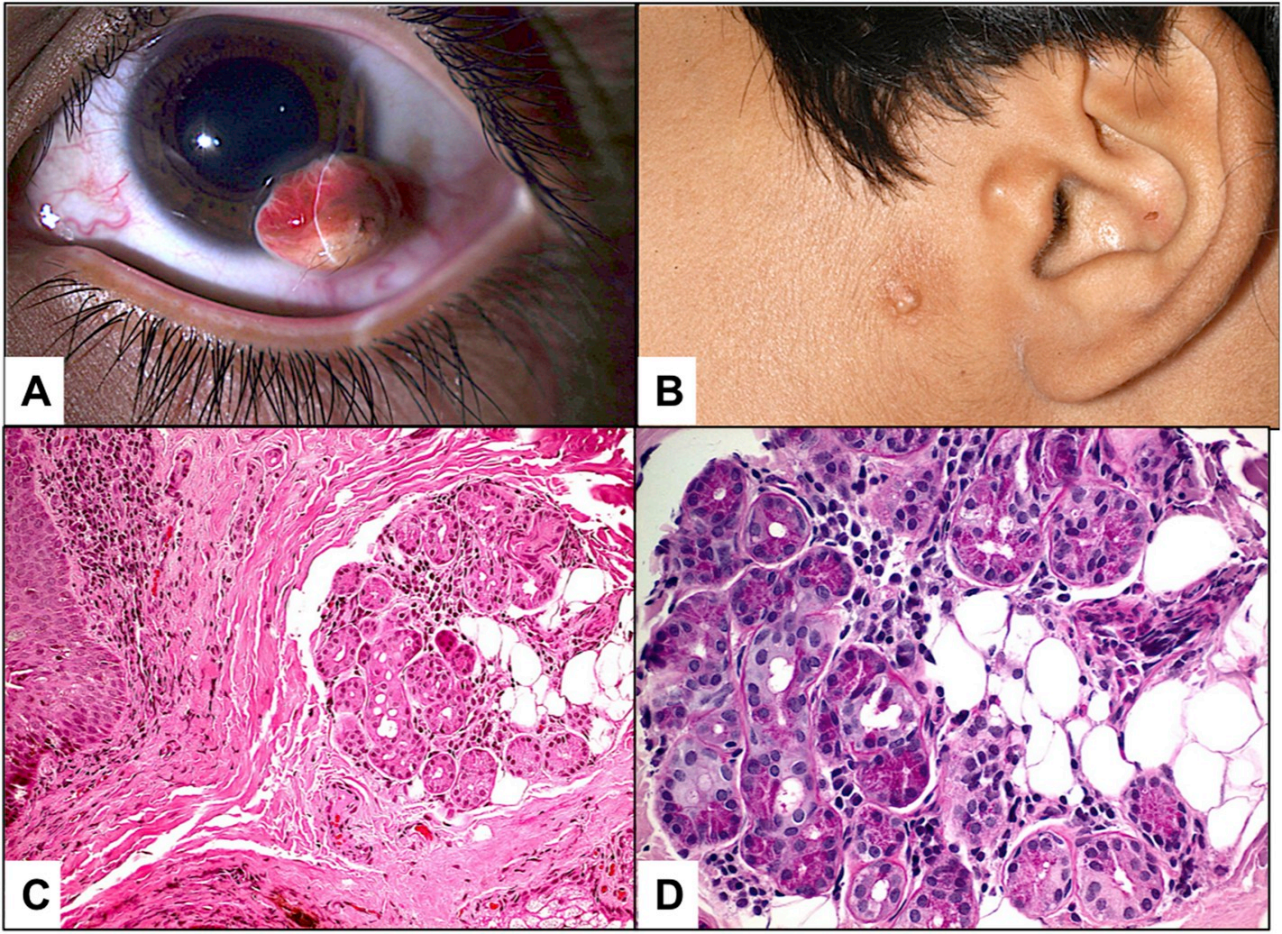
(Case 2) A. The clinical appearance of the left eye in a 14-year-old boy with Goldenhar's syndrome showing an inferotemporal epibulbar lesion since birth, but progressively increasing in size over the last year. B. Systemically the patient had one kidney, facial asymmetry, and a preauricular skin tag as shown. C. Histopathological photo of the complex choristoma excised 2 months after presentation for diagnostic and cosmetic reasons and was dome-shaped, lined by epidermal-like epithelium with pilosebaceous units, prominent wavy fibers, glandular elements and small areas of adipose tissue (Original magnification X200 Hematoxylin and eosin). D. Higher magnification of the glandular acini (Original magnification X400 Periodic acid Schiff).

**Fig. 2:**
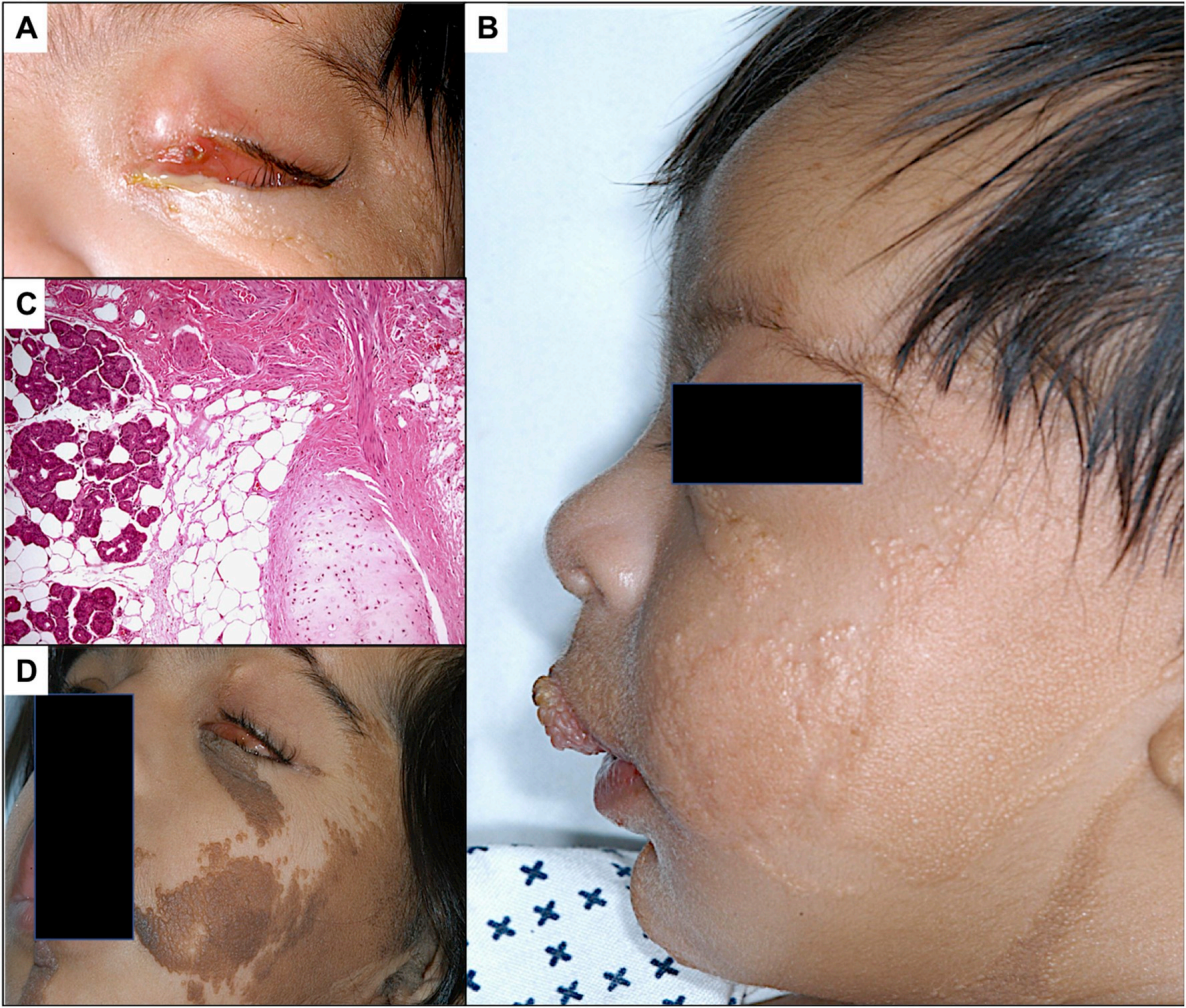
A. A 3-month-old Saudi boy with LNSS presented with a progressive lesion in the left eye since birth and no light perception. The whole left globe anteriorly was covered by a large 7-mm superior epibulbar mass preventing further view of the anterior segment and retina. B-Scan was done, which showed flat retina and choroidal calcification. B. Systematically the patient showed dysmorphic features, skin manifestations, was suffering from seizure and had confirmed developmental delay. C. The histopathologic appearance of the complex choristoma initially described as being diffuse and partially excised one month after presentation showing glandular acini, smooth muscle and cartilage embedded in prominent wavy collagen fibers and adipose tissue (Original magnification X100 Hematoxylin and eosin). D. The nevus on the face in the same patient with LNSS becoming evident by the age of 5 years.

**Fig. 3:**
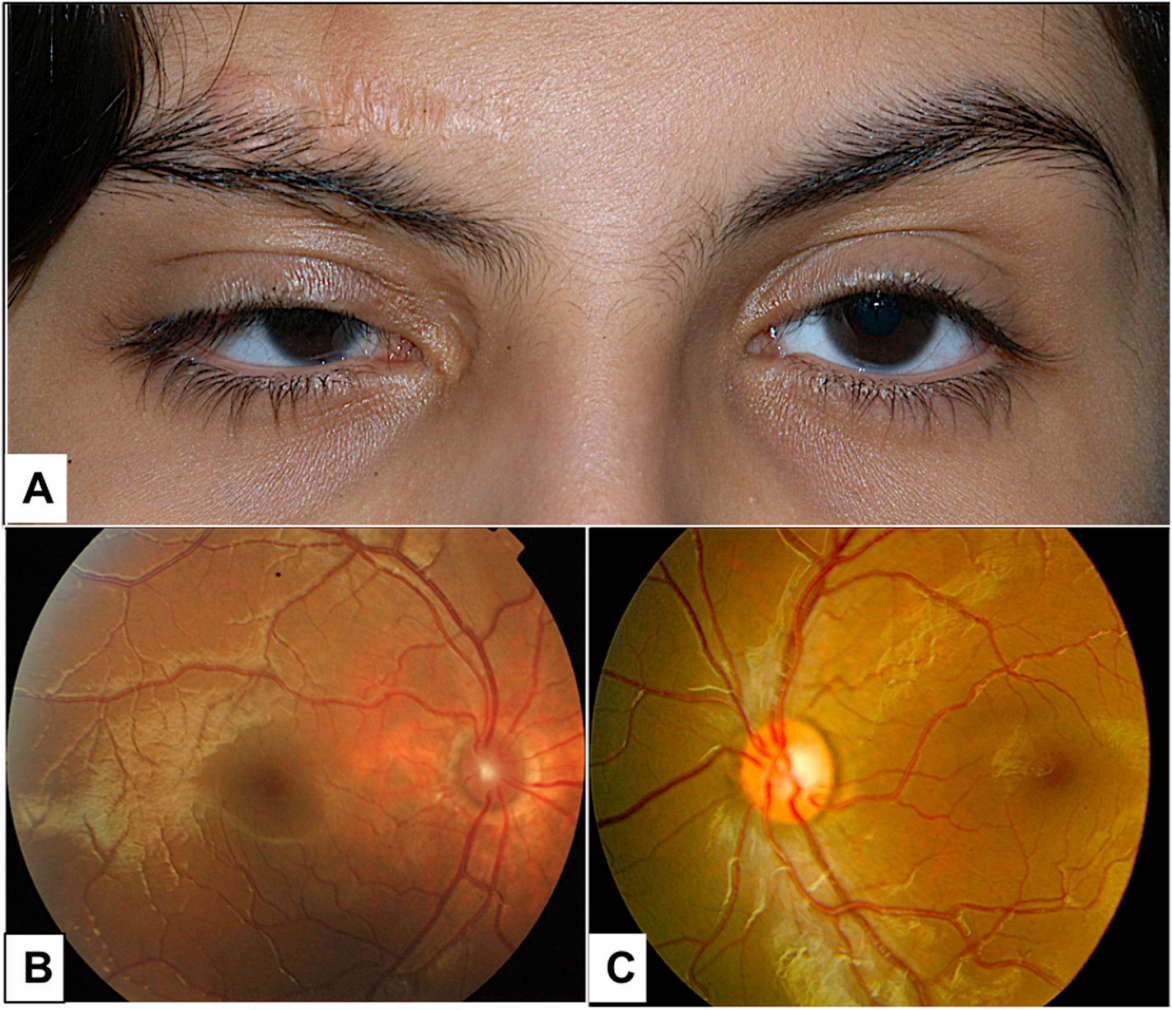
A. 7-year-old Saudi girl presented initially with a lesion in the right supero-temporal epibulbar area since birth. Systemically, the patient had abnormal skull, history of seizure, arachnoid cyst temporally and alopecia. She was also diagnosed to have intracranial lipoma and was diagnosed to have Goldenhar's syndrome and ECCL. Right eye also showed microcornea and optic nerve hypoplasia. Her refraction showed anisometropia. B. Right fundus photo showing optic nerve hypoplasia in the same patient in comparison to the normal optic nerve in the left fundus photo in C.

**Graph 1:**
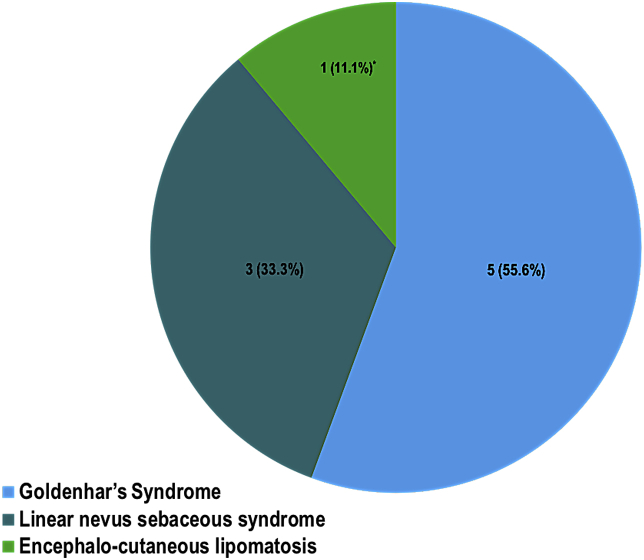
Distribution of 9 systemic associations in 8 patients with complex choristoma.

Systemic 9 associations were encountered in 8 cases of epibulbar complex choristoma (One patient had 2 associated syndromes), and none was observed in the 2 cases of osseous choristoma (Graph 1). Goldenhar's syndrome was the most common in 5/9 associations (55.6%) and these 5 cases are summarized in Table 2 with an example: (second case) in Fig. 1A–C. Similarly, the 3 cases of Linear nevus sebaceous syndrome (LNSS) are summarized in Table 3 with an example (case 2) shown in Fig. 2 A-D. All 3 cases of LNSS had the typical nevus on the face but only 2 cases were documented to have alopecia. Other systemic associations were developmental delay, macrocephaly and seizure. The ophthalmic associations other than confirmed epibulbar complex choristoma in the 3 cases of LNSS syndrome, were: lid coloboma, optic disc coloboma and choroidal calcification, which was confirmed by B scan. One case only with confirmed diagnosis of ECCL (and Goldenhar's syndrome) has been identified in our series (Fig. 3A–C).

**Table 2:** Summary of 5 cases of associated Goldenhar's syndrome and complex choristoma.

Demographic (Age/Gender)	Location of complex choristoma	Ocular association	Indication for surgery	Systemic manifestation
2 and 1/2 years Male	Temporal	NLD obstruction	Diagnostic	Mandibular hypoplasia
				Preauricular skin tag
14 years Male	Inferotemporal	None	Diagnostic + Cosmetic	Mandibular hypoplasia
				Preauricular skin tag
5 years Female	Temporal	Upper lid coloboma	Cosmetic	Preauricular skin tag
8 years Female	Infero-temporal	Upper Lid coloboma	Astigmatism	Mandibular hypoplasia
				Preauricular skin tag
				Imperforated external ear
a7 years Female	Supero-temporal	Upper lid skin tag	Cosmetic	Preauricular skin tag
		Microcornea		Alopecia (head)
		Optic nerve hypoplasia		Abnormal skull
				Seizure
				Temporal arachnoid cyst

a This case had a diagnosis of both Goldenhar's syndrome and encephalo-cranio-cutaneous lipomatosis (ECCL). NLD: Nasolacrimal duct.

**Table 3:** Summary of the 3 cases of associated LNSS and complex choristoma.

Age/Gender	Location	Ocular association(s)	Indication for surgery	Systemic manifestation
4 years Male	Nasal	Upper lid coloboma	Cosmetic + Lid repair for coloboma	Alopecia (Head)
		Restriction of up- gaze		Nevus (Face)
3 months	Superior	Dystopia	Diagnostic	Nevus (face)
		Choroidal calcification		Developmental delay
				Seizure
2 years Male	Temporal	Optic disc coloboma	Diagnostic	Macrocephaly
				Alopecia (head)
				Nevus (face)
				Developmental delay

LNSS: linear nevus sebaceous syndrome.

**Fig. 4:**
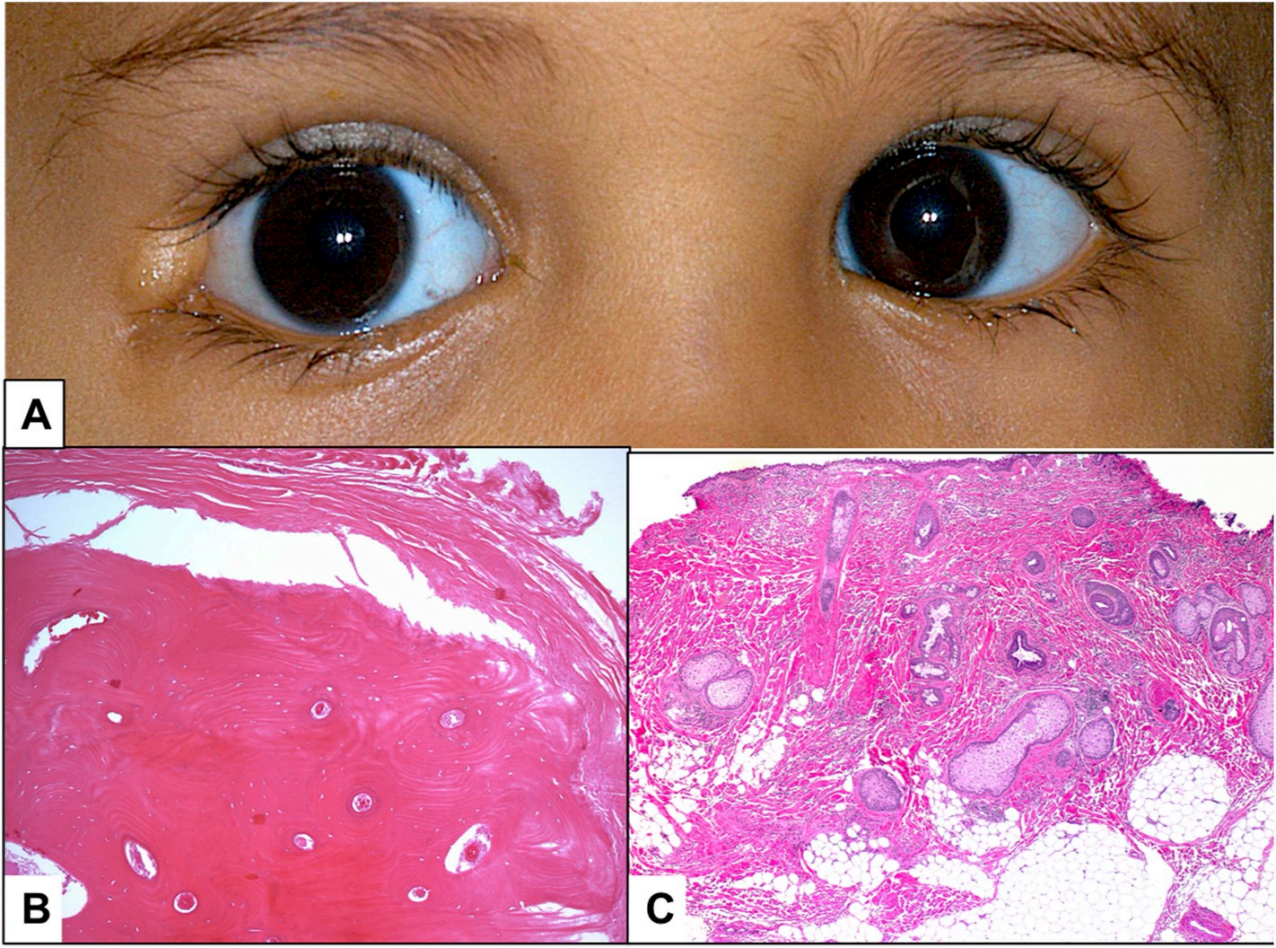
A. The clinical appearance of an osseous choristoma. B. The corresponding histopathology of the U-shaped osseous choristoma in the patient showing mature bone (Original magnification X 200 Hematoxylin and eosin). C. The typical dome-shaped appearance of epibulbar choristoma with overlying skin-like epithelium, pilosebaceous units, thick collagen fibers and fat (Original magnification X 50 Hematoxylin and eosin).

**Graph 2:**
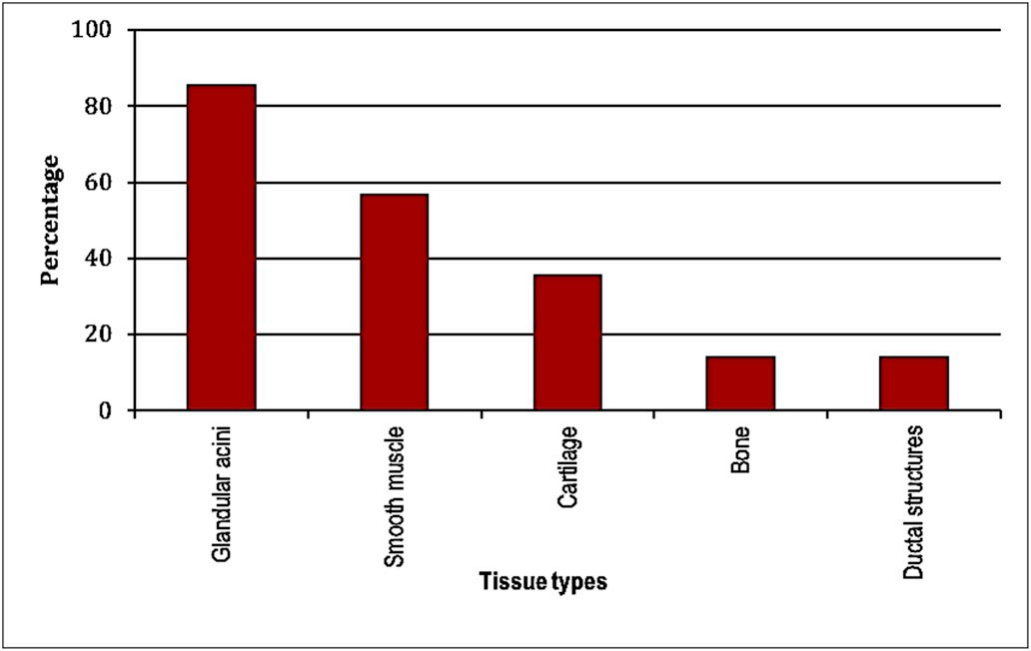
Types of tissue found in the studied 15 choristomatous lesions in order of frequency.

2 cases had confirmed histopathological diagnosis of osseous choristoma. None of the cases had any associated systemic abnormality. Both had the lesion since birth with no family history of a similar condition. One patient had an isolated osseous choristoma without any associated ophthalmic manifestations (Fig. 4A and B) and the other had an associated Coat's disease in the same eye.

All cases in our series were managed surgically and the most common indication for surgical removal was cosmetic with mean of 44.6 months between presentations to surgical intervention. None of the cases had recurrence of the lesion (defined as a re-appearing growing mass at the same epibulbar location).

All cases showed the typical epidermal-like lining with skin appendages, wavy dense collagenous fibers and adipose tissue in variable proportions (Fig. 4C), in addition to other types of tissue: glandular elements, smooth muscle fibers, cartilage, bone and ductal structures. The frequency of these different constituents is presented in Graph 2. While Table 4 shows the distribution of each type of tissue with the glandular acini being the commonest component confirmed histopathologically in 12/13 patients. We also studied the correlation between each component to 2 variables: the location and the size of the choristoma, where presence of smooth muscle component was found to be significantly associated with larger choristoma size (*p = 0.042*).

**Table 4:** Relation between average size of the choristoma and the presence of each choristomatous component.

Tissue type	Size (mm) Mean ± SD	*P value*
Glandular acini		
Yes (n = 12)	11.6 ± 5.5	0.191
No (n = 3)	8.0 ± 3.0	
Smooth muscle		
Yes (n = 8)	13.3 ± 5.3	0.042^a^
No (n = 7)	8.2 ± 3.9	
Cartilage		
Yes (n = 5)	10.6 ± 1.7	0.853
No (n = 10)	11.1 ± 6.4	
Bone		
Yes (n = 2)	8.0 ± 4.2	0.347
No (n = 13)	11.4 ± 5.3	

a Statistically significant at 5% level of significance.

## 3 Discussion

In this study, 120 Saudi cases were included, and corresponding reports were filed within a period of 16 years. Only 10.8% were of the complex type and 1.7% was of the osseous type confirming the rarity of these lesions. Choristomas are classified as congenital lesions, nevertheless, they could clinically develop later in life. This has been evident in our study since the mean age at presentation in our study was 7.6 years. Although the studied type of lesion is typically known to be stationary, previous reports have documented subjective and objective progression of these tumors close to puberty [3,11]. The present study showed similar tendency, where 40% of the studied cases showed progression around puberty age. Fig. 1 represents a 14-year-old case. There was no significant sex tendency.

The choristoma (of both types) was located in the temporal bulbar area near the limbus in more than 2 thirds of the cases and it was affecting the vision in less than one third. Complex epibulbar choristoma usually presents with other ophthalmic manifestation, and this has been further confirmed in 11 patients with lid coloboma being the most common association followed by optic nerve (ON) anomalies, which have included ON coloboma, and ON hypoplasia, then other ocular anomalies such as choroidal calcification, and microcornea. All these have been reported before. On the other hand, osseous choristoma considered the least frequent form of epibulbar choristomas, occupies 5–10 mm behind the limbus. Normally, they appear isolated and positioned in the supero-temporal quadrant, however, they can occasionally be present on other locations on the surface of the eye [[12], [13], [14]]. A pioneer in ophthalmology, Albrecht Von Graefe was the first to describe choristomas in1863, later on in 1964, Beckman and Sugar used the terminology of “osseous choristoma”, as it is recognized currently [7,15,16]. Nowadays, and according to our PubMed-based literature research, 65 documented reports only were found since then. In one of these works, in a clinical survey done by Shields and co-authors, in 1643 conjunctival lesions it was possible to identify 5% of osseous choristoma cases out of all choristomas, which was present in 1% of all the studied conjunctival lesions [17]. Osseous choristomas are usually unilateral, however, bilateral cases were also reported, and moreover they can also be associated with systemic diseases, although not the majority of the cases [6,18,19].

Interestingly, one of our 2 cases with osseous choristoma did not have an isolated lesion but had an associated Coat's disease in the same eye, which has not been previously reported to the best of our knowledge.

Systemic association was found in 8 of our complex choristoma patients and Goldenhar's syndrome was the most common -as expected-in more than half of the patients followed by LNSS in 3 cases (organoid nevus syndrome) and a single case of ECCL.

Oculo-auriculo-vertebral spectrum (OAVS) is a developmental complex craniofacial syndrome that primarily affects the structures of head and neck that are derived from the first two branchial arches and the intervening first pharyngeal pouch and brachial cleft during embryogenesis [20,21]. The characteristic findings of this syndrome include: epibulbar dermoid, ear anomalies, hemifacial microsomia, and vertebral anomalies. Goldenhar's syndrome, lateral facial dysplasia, and hemifacial microsomia are often used as alternative terms to indicate similar syndromes sharing the presence of choristoma as part of the same clinical series, with difference only in the phenotype severity [22,23]. In 1993, Kobrynski L and co-workers reported a case of an infant, associating the Trisomy 22, facio-auriculo-vertebral (Goldenhar) sequence with complex choristoma [24].

Different rare systemic syndromes associated to choristomas such as LNSS [25]. LNSS is a linear sebaceous nevus with an extensive scope of abnormalities that includes the central nervous system (CNS). This condition causes convulsions, mental retardation as well as cerebral and cerebellar hypoplasia. Also, LNSS can present with the following variety of choristomas: conjunctival simple or complex choristomas, posterior scleral cartilaginous choristoma and eyelid coloboma. Organoid nevus syndrome shows fundus lesions that have been read as choroidal coloboma and osteoma but in one case, it has been documented histopathologically to be a cartilaginous choristoma [[26], [27], [28], [29], [30]]. Of the 3 cases of organoid nevus syndrome in our series, all had nevus in the face but only 2 cases were documented to have alopecia. Other systemic associations found in our study were developmental delay, macrocephaly and seizure.

Encephalo-cranio-cutaneous lipomatosis (ECCL), or Fishman or Heberland syndrome, is another rare congenital neuro-cutaneous illness that particularly affect tissues derived from ecto-mesodermal elements, such as the skin, CNS and the eye [31,32]. classically, ECCL has a triad of cutaneous, ocular and CNS abnormalities. The cutaneous anomalies include nevus psiloliparus, subcutaneous lipoma in the fronto-temporal region, focal dermal hypoplasia or aplasia, small nodular tag on the eyelid or at outer canthal area. While some ocular abnormalities are reported to be choristomas, colobomas, corneal or anterior chamber deformities, and globe calcification, and finally the CNS abnormalities. Those cases are characterized as lipomas, anomalous intracranial vessels, hemisphere degeneration (complete or partial), asymmetrical dilatation of the ventricles or hydrocephalus, arachnoid cyst, porencephalic cyst, and calcification [[33], [34], [35]].

Our only case of combined ECCL and Goldenhar's syndrome (demonstrated in Fig. 3) had abnormal skull, seizure, temporal arachnoid cyst, alopecia and history of excised intracranial lipoma. The patient's ocular findings included right upper lid skin tag, microcornea and optic nerve hypoplasia. According to literature review there was no reported cases of ECCL associated with Goldenhar's syndrome.

Histopathologically, the choristoma in the epibulbar area will be coated by skin-like epithelium showing dense fibrous tissue and depending on other factors, different amounts of adipocytes. In the complex epibulbar choristoma, other cells can be observed including: glandular acini, smooth muscle fibers, cartilage, bone and rarely myxomatous tissue [5,36]. The osseous choristoma is typically composed of well developed, compact bone, in which the osteocytes would look histopathologically normal with harversian canals present [12].

Interestingly upon correlation of the choristomatous component and location/size of the lesion, our data analysis showed that the presence of smooth muscle was significantly associated with a larger size choristoma (*p = 0.042*).

Choristomas can be managed when choristomas are accompanied by symptoms or they became a concern to the patient and family, surgical removal would be an option. In our study, all cases were managed surgically (for diagnostic, therapeutic and cosmetic reasons) by direct excision of the mass with no complications or recurrence.

## 4 Conclusions

In spite of the fact that epibulbar complex choristoma and osseous choristoma are considered by some as 2 separate entities, they are both rare and can be associated with either systemic manifestations (in the former) or ocular manifestations (in the later). No specific sex predilection has been found, most cases are documented to have the lesion since birth with possible progressive increase in size around puberty, and they are mostly temporal in location. Histopathologically, the choristomas in our series were not different than what has been reported, however, we have demonstrated that the presence of smooth muscle is significantly associated with a larger size choristoma.

Interesting ophthalmic associations were frequently encountered in our complex choristoma cases with the lid anomalies followed by ON anomalies being the commonest. The association of osseous choristoma and coat's disease in the same eye is being observed in our study for the first time. Out of the 13 cases of complex choristoma, more than half had associated syndromes with Goldenhar's syndrome being the commonest in addition to LNSS and ECCL. Our single diagnosed case of ECCL had clinical evidence of an associated Goldenhar's syndrome. Even though our sample size is small, these associations might raise the necessity for genetic studies in our community where consanguinity is common.

We recommend such genetic research for better understanding of the pathogenesis of these lesions and consideration of molecular therapy.

## Declaration statement

This study was prepared in accordance with the ethical standards of the human ethics committee at KKESH and approval from the Research department in accordance with the Helsinki Declaration. A general informed consent was taken from all cases which includes permission for anonymous use of photos and reporting. The authors have no conflict of interest or financial disclosures in relation to this work.

## Provenance and peer review

Not commissioned, externally peer reviewed.

## Conflicts of interest

The authors have no conflict of interest or financial disclosures in relation to this work.

## Funding

None.

## Ethical approval

This study was prepared in accordance with the ethical standards of the human ethics committee at KKESH and approval from the Research department in accordance with the Helsinki Declaration (RP-1664-R). A general informed consent was taken from all cases which includes permission for anonymous use of photos and reporting.

## Trial registry number

researchregistry4178.

## Author contribution

First author:Collection of Data, literature search and drafting the manuscript.

Second author: Histopathological review and overall review for editing of the manuscript.

Third author: Histopathological review and images.

## Guarantor

Dr. Hind Manaa Alkatan.
